# A super-spreader of SARS-CoV-2 in incubation period among health-care workers

**DOI:** 10.1186/s12931-020-01592-w

**Published:** 2020-12-10

**Authors:** Chaojie Wei, Yufeng Yuan, Zhenshun Cheng

**Affiliations:** 1 Department of Pulmonary and Critical Care Medicine, Zhongnan Hospital of Wuhan University, No.169 Donghu Road, Wuhan, 430071 China; 2 Department of Hepatobiliary Surgery, Zhongnan Hospital of Wuhan University, No.169 Donghu Road, Wuhan, 430071 China; 3 Wuhan Research Center for Infectious Diseases and Cancer, Chinese Academy of Medical Sciences, No.169 Donghu Road, Wuhan, 430071 China

**Keywords:** Coronavirus disease 2019, Health-care workers, Super-spreader

## Abstract

Since the coronavirus disease 2019 (COVID-19) identified in Wuhan, Hubei, China in December 2019, it has been characterized as a pandemic by World Health Organization (WHO). It was reported that asymptomatic persons are potential sources of severe acute respiratory syndrome coronavirus 2 (SARS-CoV-2) infection. We present an outbreak among health-care workers incited by a doctor who cared a patient with COVID-19 in a Hospital in Wuhan, Hubei, China, which indicates existence of super-spreader even during incubation period.

To the editor:

Since the coronavirus disease 2019 (COVID-19) identified in Wuhan, Hubei, China in December 2019, it has been characterized as a pandemic by World Health Organization (WHO). Person-to-person transmission pattern of COVID-19 is obvious [1, 2]. Moreover, it was reported that asymptomatic persons are potential sources of severe acute respiratory syndrome coronavirus 2 (SARS-CoV-2) infection [3]. Here, we present an outbreak among health-care workers incited by a doctor who cared a patient with COVID-19 in Hospital A, in Wuhan, Hubei, China, which indicates existence of super-spreader during incubation period and sustained human-to-human transmission of COVID-19.

A 66-year-old woman (patient A_1_) with a history of cholecystolithiasis presenting right epigastric pain, fever, and tenderness in the right upper abdomen was admitted by doctor B to department A for consideration of acute cholecystitis. During preoperative preparation, patient A_1_ presented progressive dyspnea. Chest computed tomography (CT) scan showed bilateral wide spread ground-glass opacity at day 6 after admission. Then, real-time reverse transcription-polymerase chain reaction (RT-PCR) of a throat swabs has been done and confirmed SARS-CoV-2 infection. Patient A_1_ was transferred immediately to isolation ward. Without realization of infectious disease, doctor B didn’t use any personal protectives when provided health care for patient A_1_.

The exposure history was shown in Fig. 1. A multiple disciplinary team meeting (MDT) was hold by department A on the third day after doctor B first exposure to patient A_1_. About 40 health-care workers (including doctor B–Q and nurse R) attended the MDT. The MDT lasted for about several hours in a closed meeting room. After the MDT, doctor F conducted a consultation for his patient. Doctor S and T from other two departments communicated with doctor F during the consultation for less than half an hour.

**Fig. 1:**
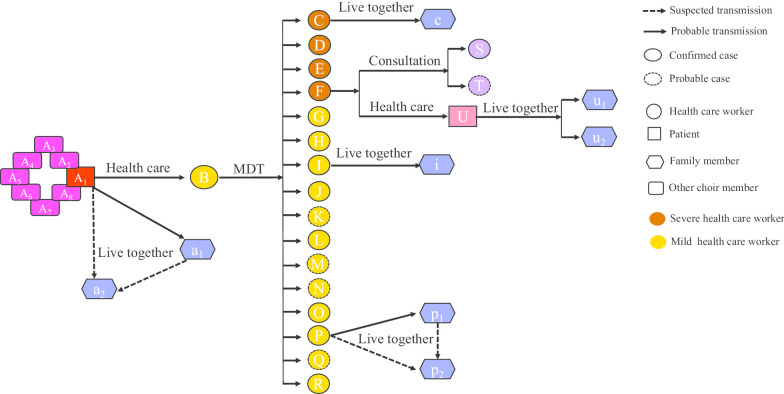
Transmission map of outbreak of COVID-19. All confirmed cases and the five probable cases linked to transmission event are shown. Putative transmissions are indicated. The letters within symbols are health-care workers, patients, family members and choir members identifiers

Within 10 days, 14 health care workers presented symptoms, such as fever, cough, fatigue, myalgia, etc. (Additional file 1: Table S1). Doctor H even felt unwell on the day after the MDT. Doctor B presented symptoms 3 days after the MDT. All 19 health-care workers had abnormal chest CT scan with ground-glass opacity and/or subsegmental areas of consolidation, but normal white blood cell counts and procalcitonin (PCT) values. Assays of SARS-CoV-2 by real-time RT-PCR from throat swabs samples were positive in 14 of 19 health-care workers (Additional file 1: Table S1). The 5 with negative nucleic acid test of SARS-CoV-2 all had abnormal chest CT scan with unilateral ground-glass opacity. The negative result of SARS-CoV-2 test may be explained by low viral load and only once nucleic acid test.

Afterwards, as shown in Fig. 1, the wife (c, i, p_1_) of doctor C, I, and P respective, the mother-in-law (p_2_) of doctor P and a patient (U) of doctor F were confirmed COVID-19. The wife (u_1_) and mother (u_2_) of patient U were also confirmed as COVID-19 later. In addition, patient A_1_ was a member of a chorus in which they often sang together. Seven members (A_2_–A_8_) of his choir were confirmed or suspected COVID-19 and two were died. The son (a_1_) of patient A_1_ was also confirmed COVID-19 as well as his cousin (a_2_).

After inquiry of all the health-care workers attended the MDT, we learnt that doctor B was the only person who was definitely exposed to the person with confirmed COVID-19 before the MDT. Therefore, doctor B was the most possible source of SARS-CoV-2 in the health-care workers attended the MDT. The possibility that some health-care workers in the same department as doctor B may be infected by doctor B during daily work cannot be precluded. It was reported that asymptomatic persons are potential sources of SARS-CoV-2 infection [3]. The MDT was hold 3 days before symptoms onset of doctor B. Doctor B was in incubation period when attended the MDT. Whereas, doctor B was yet induced transmission of SARS-CoV-2 to 16 health-care workers. Thus, a person infected SARS-CoV-2 may be a ‘super-spreader’ even during incubation period. Indeed, the presence of ‘super-spreaders’ of COVID-19 cannot be precluded in the large clinical research of Dr. Zhong’s group [4]. Phenomenon of super-spreading was observed in SARS and MERS [5]. In addition, the third and fourth generation of COVID-19 was detected in this case, which indicated possible sustained human-to-human transmission of COVID-19.

Among the 14 health-care workers who presented symptoms, the median incubation time was 4.0 days (IQR 2.0–6.0). However, the shortest incubation time observed in this case was less than 1 day, which is consistent to the recent large clinical research of Dr. Zhong’s group [4].

## Supplementary information

**Additional file 1:** **Table S1.** Characteristics of patient A_1_ and health care workers with confirmed or suspected COVID-19.

## Data Availability

The datasets used and/or analysed during the current study are available from the corresponding author on reasonable request.

## Abbreviations

COVID-19: Coronavirus disease 2019
SARS-CoV-2: Severe acute respiratory syndrome coronavirus 2
WHO: World Health Organization
CT: Computed tomography
RT-PCR: Reverse transcription-polymerase chain reaction
MDT: Multiple disciplinary team meeting
PCT: Procalcitonin
IQR: Interquartile range
