# Correction to: A super-spreader of SARS-CoV-2 in incubation period among health-care workers

**DOI:** 10.1186/s12931-021-01626-x

**Published:** 2021-01-19

**Authors:** Chaojie Wei, Yufeng Yuan, Zhenshun Cheng

**Affiliations:** 1 Department of Pulmonary and Critical Care Medicine, Zhongnan Hospital of Wuhan University, No.169 Donghu Road, Wuhan, 430071 China; 2 Department of Hepatobiliary Surgery, Zhongnan Hospital of Wuhan University, No.169 Donghu Road, Wuhan, 430071 China; 3 Wuhan Research Center for Infectious Diseases and Cancer, Chinese Academy of Medical Sciences, No.169 Donghu Road, Wuhan, 430071 China

## Correction to: Respir Res (2020) 21:327 http://doi.org/10.1186/s12931-020-01592-w

Following publication of the original article [1], we were notified that a funding note needs to be added to the Funding section: "Ministry of Science and Technology of the People's Republic of China (Grant No. 2020YFC0845500)."

The original paper has been corrected.
