# Supplementary material for: A super-spreader of SARS-CoV-2 in incubation period among health-care workers
Source: Respir Res. 2020 Dec 10;21:327. doi: 10.1186/s12931-020-01592-w (PMC7727779; doi:10.1186/s12931-020-01592-w)
Supplement: Supplementary file 1 — Additional file 1: Table S1. Characteristics of patient A1 and health care workers with confirmed or suspected COVID-19. [file 12931_2020_1592_MOESM1_ESM.docx]

**Additional file**

**Table S1 Characteristics of patient A_1_ and health care workers with confirmed or suspected COVID-19**

| Characteristics | Patient | Health care worker | | | | | | | | | | | | | | | | | | |
| --- | --- | --- | --- | --- | --- | --- | --- | --- | --- | --- | --- | --- | --- | --- | --- | --- | --- | --- | --- | --- |
|  | A_1_ | B | C | D | E | F | G | H | I | J | K | L | M | N | O | P | Q | R | S | T |
| Age(yr) | 66 | 61 | 51 | 43 | 66 | 42 | 53 | 33 | 57 | 37 | 49 | 61 | 49 | 51 | 53 | 51 | 52 | 30 | 45 | 33 |
| Sex | F | M | M | M | M | M | M | M | M | M | M | M | M | M | M | M | M | F | M | M |
| Comorbidities | ×^*^ |  |  |  | ×^*^ | ×^*^ |  |  |  |  |  |  |  | ×^*^ |  | ×^*^ |  |  |  |  |
| Type of exposure | | | | | | | | | | | | | | | | | | | | |
| Health care |  | × |  |  |  |  |  |  |  |  |  |  |  |  |  |  |  |  |  |  |
| MDT |  |  | × | × | × | × | × | × | × | × | × | × | × | × | × | × | × | × |  |  |
| Consultation |  |  |  |  |  |  |  |  |  |  |  |  |  |  |  |  |  |  | × | × |
| Days from exposure to onset of symptoms | NA | 6 | 1 | <1 | 5 | 2 | NS | 4 | 1 | 6 | NS | 2 | NS | 6 | 4 | 3 | NS | 10 | 6 | NS |
| Symptoms at admission | | | | | | | | | | | | | | | | | | | | |
| Fever | × |  | × | × |  | × |  | × |  |  |  |  |  |  | × | × |  |  | × |  |
| Chill |  |  |  |  |  | × |  | × |  |  |  |  |  |  |  |  |  |  |  |  |
| Cough |  | × | × |  |  |  |  |  | × | × |  | × |  | × |  | × |  |  | × |  |
| Sputum production |  | × | × |  |  |  |  |  |  |  |  | × |  |  |  |  |  |  |  |  |
| Myalgia |  |  |  |  | × |  |  | × |  |  |  |  |  |  |  |  |  | × |  |  |
| Fatigue |  | × |  | × |  | × |  | × |  | × |  |  |  | × |  |  |  |  |  |  |
| Pharyngalgia |  | × |  |  |  |  |  | × |  |  |  |  |  |  |  |  |  |  |  |  |
| Dyspnea |  |  |  |  |  |  |  |  | × |  |  |  |  |  |  |  |  |  |  |  |
| Diarrhea |  | × |  |  |  |  |  |  |  |  |  |  |  |  |  |  |  |  |  |  |
| Nausea |  |  |  |  |  |  |  |  |  |  |  |  |  |  |  |  |  | × |  |  |
| Dizziness |  |  |  |  |  |  |  |  |  |  |  |  |  |  |  |  |  | × |  |  |
| Right epigastric pain | × |  |  |  |  |  |  |  |  |  |  |  |  |  |  |  |  |  |  |  |
| Laboratory results | | | | | | | | | | | | | | | | | | | | |
| WBC x10^9^/L | 4.52 | 3.02 | 5.3 | 6.79 | 2.72 | 7.92 | 5.97 | 2.94 | 4.36 | 6.13 | NT | 4.22 | 8.95 | 5.55 | 4.87 | 4.47 | NT | 2.22 | 3.34 | 6.81 |
| Neu x10^9^/L | 3.42 | 1.40 | 3.37 | 4.98 | 1.76 | 6.04 | 3.95 | 1.18 | 2.38 | 3.28 | NT | 2.66 | 4.84 | 2.96 | 3.45 | 2.49 | NT | 1.16 | 1.43 | 3.76 |
| Lym x10^9^/L | 0.78 | 1.08 | 1.29 | 1.11 | 0.52 | 1.30 | 1.24 | 1.45 | 1.39 | 2.24 | NT | 1.00 | 3.06 | 1.52 | 1.07 | 1.18 | NT | 0.78 | 1.41 | 2.16 |
| Mon x10^9^/L | 0.30 | 0.34 | 0.50 | 0.68 | 0.43 | 0.54 | 0.76 | 0.31 | 0.55 | 0.54 | NT | 0.54 | 0.64 | 1.03 | 0.34 | 0.72 | NT | 0.27 | 0.46 | 0.75 |
| PLT x10^9^/L | 131 | 198 | 182 | 328 | 118 | 178 | 205 | 178 | 129 | 189 | NT | 162 | 231 | 127 | 148 | 160 | NT | 117 | 152 | 221 |
| PCT | 0.13 | <0.05 | <0.05 | <0.05 | <0.05 | <0.05 | NT | NT | <0.05 | <0.05 | NT | <0.05 | NT | <0.05 | NT | <0.05 | NT | <0.05 | <0.05 | NT |
| CRP | NT | 1.6 | 2.7 | 22 | 17.6 | 23.7 | NT | NT | 8.6 | 8.0 | NT | 20.7 | NT | 8.9 | 1.72 | 18.4 | NT | 5.5 | 3.5 | NT |
| Involvement of chest CT scan | | | | | | | | | | | | | | | | | | | | |
| Bilateral | × |  | × | × | × | × |  | × | × | × |  | × |  |  | × | × |  |  |  |  |
| Unilateral |  | × |  |  |  |  | × |  |  |  | × |  | × | × |  |  | × | × | × | × |
| SARS-CoV-2 RT-PCR test | **+** | **+** | **+** | **+** | **+** | **+** | **+** | **+** | **+** | **+** | **-** | **+** | **-** | **-** | **+** | **+** | **-** | **+** | **+** | **-** |
| Other respiratory etiology RT-PCR test | | | | | | | | | | | | | | | | | | | | |
| Influenza A virus | **-** | **-** | **-** | **-** | **-** | **-** | **-** | NT | **-** | **-** | NT | **-** | NT | **-** | NT | NT | NT | NT | NT | NT |
| Influenza B virus | **-** | **-** | **-** | **-** | **-** | **-** | **-** | NT | **-** | **+** | NT | **-** | NT | **-** | NT | NT | NT | NT | NT | NT |
| Respiratory syncytial virus | **-** | **-** | **-** | **-** | **-** | **-** | **-** | NT | **-** | **+** | NT | **-** | NT | **-** | NT | NT | NT | NT | NT | NT |
| Parainfluenza virus | **+** | **-** | **-** | **-** | **-** | **-** | **-** | NT | **-** | **-** | NT | **-** | NT | **-** | NT | NT | NT | NT | NT | NT |
| Adenovirus | **-** | **-** | **-** | **-** | **-** | **-** | **-** | NT | **-** | **-** | NT | **-** | NT | **-** | NT | NT | NT | NT | NT | NT |
| Mycoplasma pneumoniae | **-** | **-** | **-** | **-** | **-** | **-** | **-** | NT | **-** | **-** | NT | **-** | NT | **-** | NT | NT | NT | NT | NT | NT |
| Chlamydia pneumoniae | **-** | **-** | **-** | **-** | **-** | **-** | **-** | NT | **-** | **-** | NT | **-** | NT | **-** | NT | NT | NT | NT | NT | NT |

1. Abbreviation: F: Female; M: Male; MDT: Multi-Disciplinary Team meeting; WBC: White blood cell count; Neu: Neutrophil count; Lym: Lymphocyte count; Mon: Monocyte count; PLT: Platelets; PCT: Procalcitonin; CRP: C-reactive protein; ×: with indicating characteristics; Space: without indicating characteristics; NA: not available; NS: No symptoms; NT: No test; +: Positive; -: Negative

2. * Details of comorbidities: A_1_: cholecystolithiasis; Hypertension; Diabetes; E: Impaired glucose tolerance; F: Allergic purpura; Lymph node tuberculosis; N: Hyperlipidemia; P: Hyperuricemia
